# Engineering 2D Cu-composed metal–organic framework nanosheets for augmented nanocatalytic tumor therapy

**DOI:** 10.1186/s12951-022-01250-x

**Published:** 2022-02-04

**Authors:** Shangwen Zhuang, Huijing Xiang, Yixin Chen, Lulu Wang, Yu Chen, Jun Zhang

**Affiliations:** 1 Department of Radiology, Huashan Hospital, State Key Laboratory of Medical Neurobiology, Fudan University, Shanghai, 200040 People’s Republic of China; 2 Materdicine Lab, School of Life Sciences, Shanghai University, Shanghai, 200444 People’s Republic of China

**Keywords:** Metal–organic framework, Nanocatalytic therapy, Fenton reaction, Tumor therapy, Nanomedicine

## Abstract

The engineered nanoformulation that can be activated by intracellular tumor microenvironment, including acidic pH, overexpressed H_2_O_2_, and high concentration of glutathione (GSH), features high efficacy to eradicate tumor cells with the intrinsic specificity and therapeutic biosafety. However, the relatively slow reaction rate of traditional Fe^2+^-mediated Fenton reaction induces the low production amount of reactive oxygen species (ROS) and subsequently the limited therapeutic outcome against tumors. Here, we established Cu (II)-based two-dimensional (2D) metal–organic framework (MOF) nanosheets as a distinct chemoreactive nanocatalyst for GSH-triggered and H_2_O_2_-augmented chemodynamic therapy (CDT), depending on the “AND” logic gate, for significant tumor suppression. After internalization by tumor cells, the MOF catalytic nanosheets reacted with local GSH for inducing GSH consumption and reducing the Cu^2+^ into Cu^+^. Subsequently, abundant hydroxyl radicals (·OH) generation was achieved via Cu^+^-mediated Fenton-like catalytic reaction. The dual effects of ·OH production and GSH depletion thus enhanced ROS production and accumulation in tumor cells, leading to significant cellular apoptosis and tumor inhibition, which was systematically demonstrated in both 4T1 and MDA-MB-231 tumor models. Therefore, GSH and H_2_O_2_, serve as an “AND” logic gate to trigger the Cu^+^-mediated Fenton-like reaction and reduce GSH level for augmented CDT with high therapeutic specificity and efficacy, thus inducing cellular apoptosis primarily through ferroptosis at the RNA sequence level.

**Graphical Abstract:**

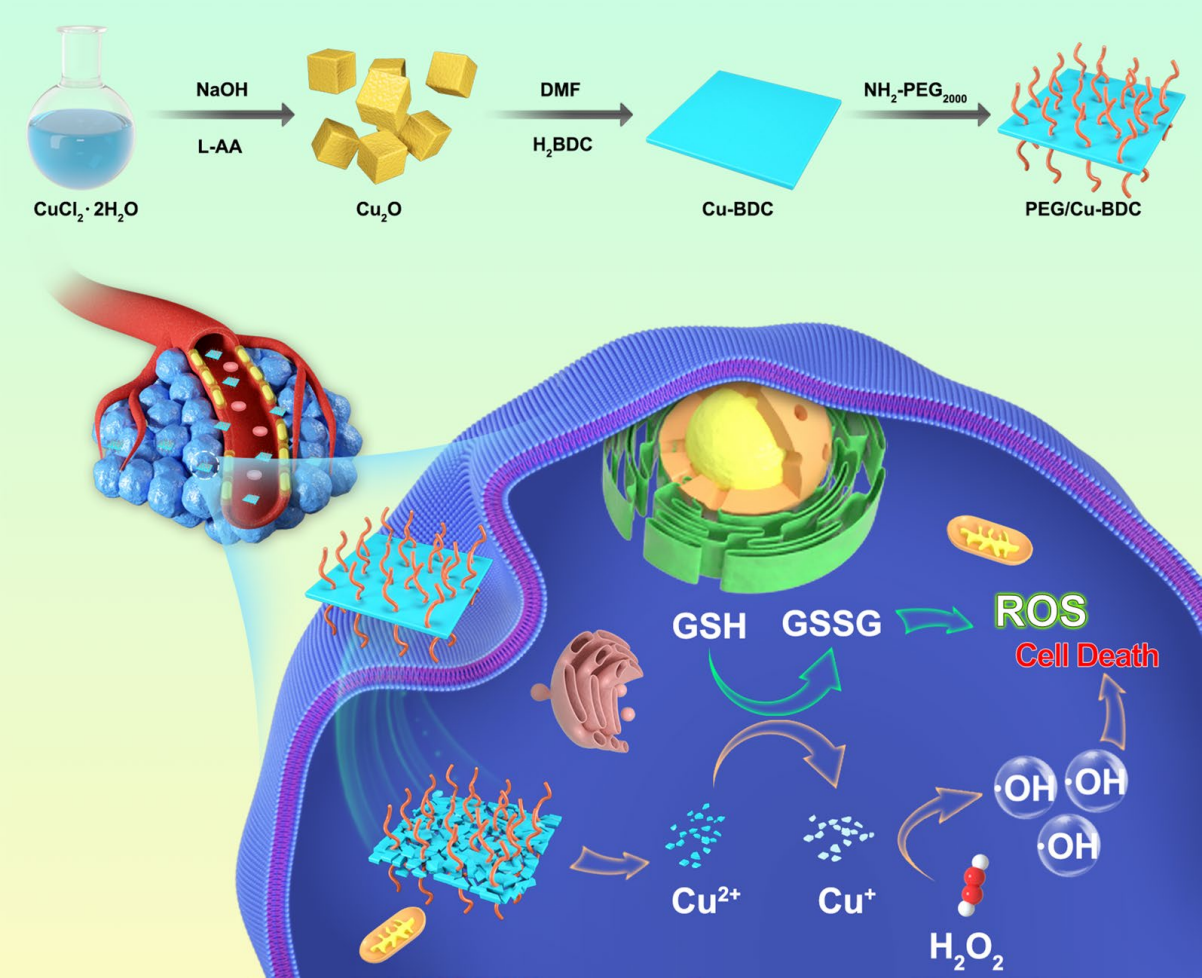

**Supplementary Information:**

The online version contains supplementary material available at 10.1186/s12951-022-01250-x.

## Introduction

The initiated production of toxic reactive oxygen species (ROS) with the aid of nanoformulations has been exploited as an effective therapeutic modality for tumor treatment [1–5]. In particular, chemodynamic therapy (CDT) typically utilizes ferrous ions (Fe^2+^) to catalyze the production of highly oxidative hydroxyl radicals (**·**OH) [6–9] through Fenton or Fenton-like chemical reactions with excess hydrogen peroxide (H_2_O_2_) in tumor microenvironment (TME) [10–12]. In comparison with other external energy input-initiated therapeutic strategies, CDT activated by the intrinsic chemical energy conversion in tumorous tissues rather than external energy circumvents the rapid attenuation in energy input during the therapeutic processes [13]. Furthermore, the transition-metal-containing nanocatalysts respond to the unique TME characteristics for CDT [14, 15], featuring low invasiveness and high treatment specificity. However, the Fe^2+^-mediated Fenton reactions proceed effectively in highly acidic milieu (pH 2–4) [16]. In addition, even at the desirable reaction surroundings, the relatively slow reaction rates of Fe^2+^-mediated Fenton reactions contribute to low capability in inducing ROS generation. Therefore, it is highly desirable and necessary to engineer the iron-free transition-metal-containing nanoformulations [17, 18] that feature a high therapeutic specificity and favorable catalytic performance in weakly acidic TME for efficient CDT-based cancer treatment.

By contrast, the Cu-based Fenton-like nanocatalysts are preliminarily demonstrated to be the potential candidates for CDT because of their high efficiency in weakly acidic TME [19, 20]. Especially, the reaction rate of Cu^+^ was calculated to be 1 × 10^4^ M^−1^ s^−1^ [21, 22], almost 160-fold than that of Fe^2+^ (~ 63 M^−1^ s^−1^) [23, 24]. However, Cu^+^ is extremely unstable and easily oxidized into Cu^2+^ attributing to the low redox potential of Cu^2+^/Cu^+^ [25]. Moreover, excess free Cu^+^ may cause severe systemic toxicity [26–28]. In addition, the ROS produced through Cu^+^-catalyzed Fenton-like reaction would be reduced and quenched by excessive reductive molecules in tumorous cells, which thus lowers the therapeutic efficacy of CDT. Therefore, it is crucially significant to spatiotemporally confine the nanoformulations at tumor regions to avoid the production of free Cu^+^ during the blood circulation [29]. Furthermore, it is conceived that we can fabricate Cu^2+^-containing nanoformulations with high stability in physiological media, which can be specifically reduced to Cu^+^ at tumor sites by excess glutathione (GSH) in TME [30–35]. Subsequently, efficient ROS production can be achieved through Cu^+^-catalyzed Fenton-like reaction with overexpressed H_2_O_2_ in tumorous tissues.

**Fig. 1:**
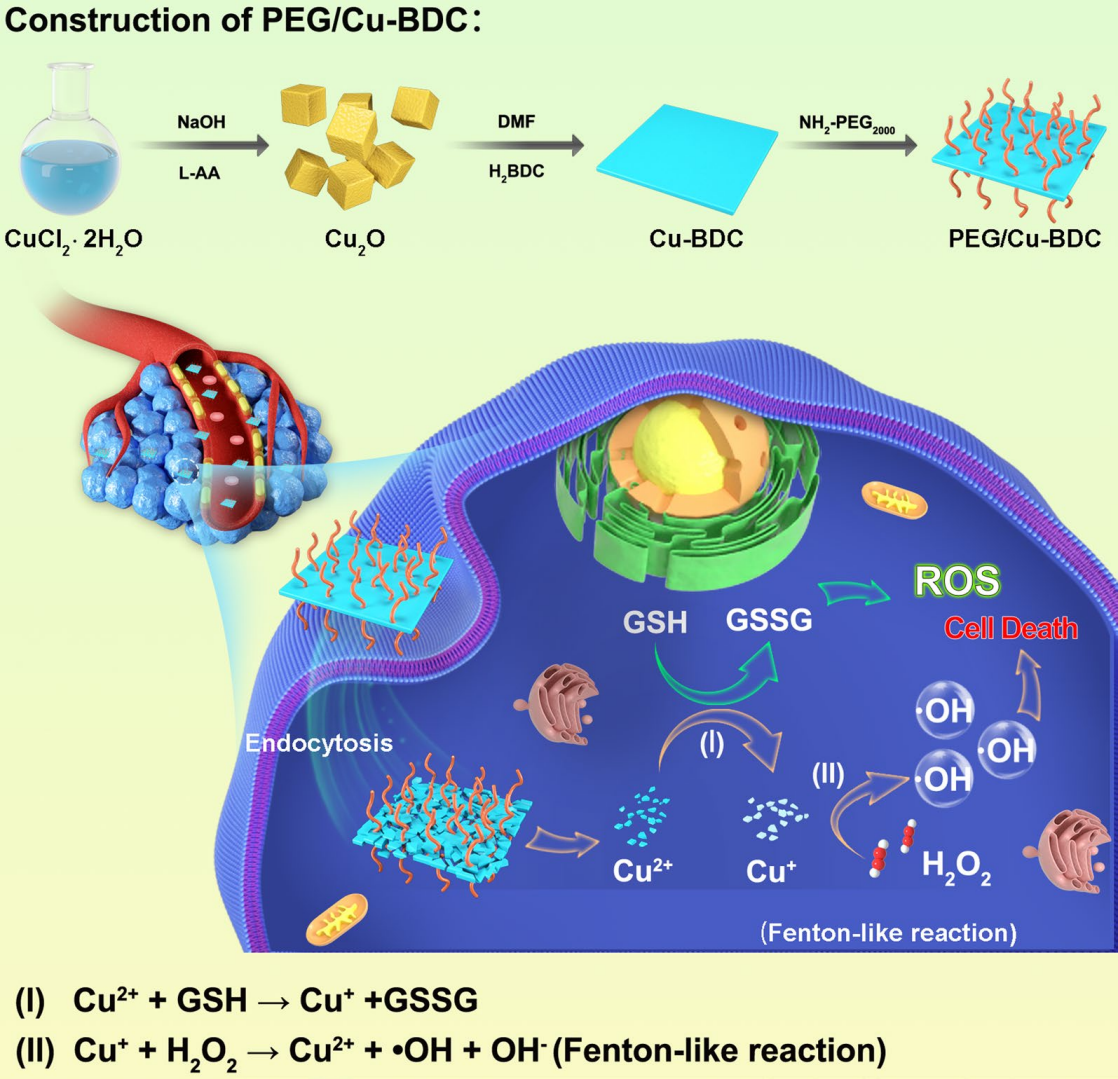
The fabrication of PEG/Cu-BDC nanocatalyst and its therapeutic application for augmented CDT through the activation of Cu^+^-catalyzed Fenton-like reaction and reduction of GSH levels

As one type of burgeoning porous nanoformulations, nanosized metal–organic frameworks (MOFs) have been extensively utilized in catalysis, biosensing, and theranostic applications [36–40], attributing to their unique superiorities of large specific surface area as well as structural tunability and diversity. Considering the high specific surface area and active centers of nanosized MOF, we assumed that a rationally tailored nanosized MOF with dual functions should be qualified to decrease the GSH level and successively activate Cu^+^-mediated Fenton-like reaction, thus augmenting the therapeutic efficacy of CDT depending on the “AND” logic gate. Here, we designed and constructed a nanocatalyst, i.e., Cu (II)-based nanosized MOF (PEG/Cu-BDC; BDC^2−^ = 1,4-benzenedicarboxylate), for achieving the augmented and TME-activated CDT against tumors. Once endocytosis by tumor cells, the released Cu^2+^ in PEG/Cu-BDC MOF was reduced to Cu^+^ along with the transformation from GSH to oxidized GSH (GSSG) (Eq. 1), followed by H_2_O_2_ consumption, ·OH generation, and oxidation from Cu^+^ to Cu^2+^ through Fenton-like reaction (Eq. 2). The double effects of ·OH generation and GSH consumption elevate the ROS level in tumor cells, inducing the effective cell apoptosis and tumor suppression. Therefore, GSH and H_2_O_2_, overexpressed in tumorous tissues, serve as an “AND” logic gate to activate the Cu^+^-catalyzed Fenton-like reaction and decrease the GSH level for augmented CDT with high therapeutic efficiency and tumor specificity. This work introduces a novel copper nanosized metal–organic framework responsive to the TME, which may have immense potential in chemodynamic cancer therapy (Fig. 1).

1 $${\text{Cu}}^{{{2} + }} + {\text{ GSH }} \to {\text{ Cu}}^{ + } + {\text{GSSG}}{.}$$

2 $${\text{Cu}}^{ + } + {\text{ H}}_{{2}} {\text{O}}_{{2}} \to {\text{ Cu}}^{{{2} + }} + \, \cdot {\text{OH }} + {\text{ OH}}^{ - } .$$

## Results and discussion

### Design, synthesis and characterization of 2D PEG/Cu-BDC nanocatalysts

**Fig. 2:**
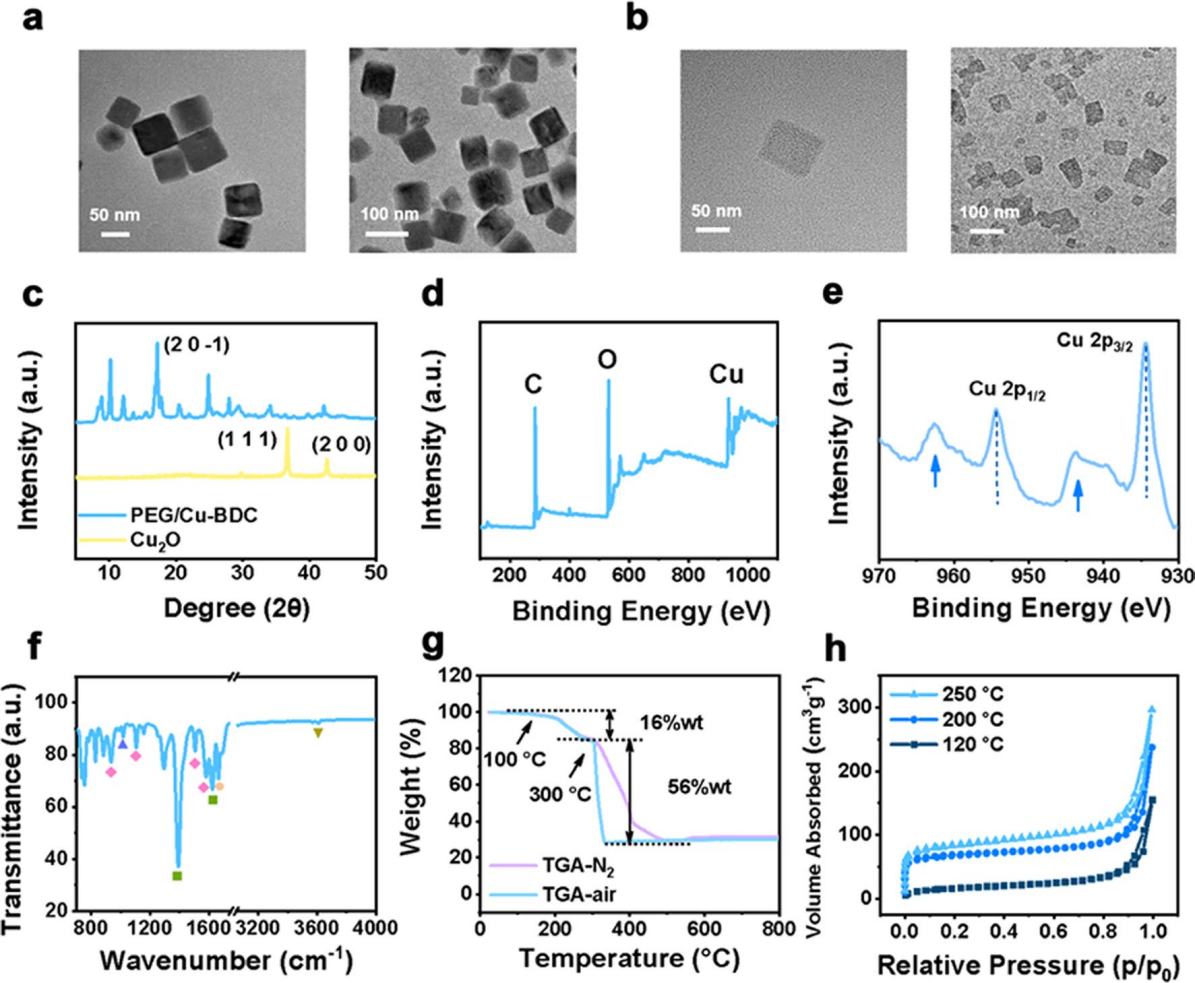
Structure, morphology, and composition characterization of PEG/Cu-BDC. **a** TEM images of Cu_2_O. **b** TEM images of PEG/Cu-BDC. **c** XRD patterns of Cu_2_O and PEG/Cu-BDC. **d** XPS spectrum of the PEG/Cu-BDC. **e** XPS spectrum of Cu 2p in PEG/Cu-BDC. **f** FTIR spectrum of PEG/Cu-BDC. **g** TGA curves of PEG/Cu-BDC in air and N_2_. **h** N_2_ absorption–desorption isotherms of PEG/Cu-BDC activated at 120, 200, and 250 ℃ for 12 h prior to the tests, respectively

There are two main procedures in the synthesis of PEG/Cu-BDC, with the color change from transparent to yellow in the first step owing to the generation of Cu_2_O and yellow to blue in the conversion process of Cu^+^ to Cu^2+^ [41]. The morphology, composition, structure, and porosity of the as-obtained Cu-BDC nanosheets were confirmed by diverse characterization techniques. As observed by transmission electron microscopy (TEM), Cu_2_O showed cubic morphology with a mean size of 60 nm, whereas PEG/Cu-BDC nanosheets were square with a diameter of approximately 90 nm (Fig. 2a, b). As for X-ray diffraction (XRD) patterns, the disappearing of Cu_2_O peaks and emerging of the main (2 0 − 1) crystallographic planes in Fig. 2c illustrated the complete conversion of Cu_2_O to Cu-BDC nanosheets. In addition, the X-ray photoelectron spectroscopy (XPS) analysis affirmed that the Cu-BDC nanosheets were composed of Cu, C, and O elements (Fig. 2d). The Cu 2p core peak of Cu-BDC nanosheets revealed two main components at 954.4 eV and 934.4 eV, with a satellite peak located at 962.6 eV and 943.7 eV, respectively, validating the presence of Cu^2+^ in the structure of Cu-BDC nanosheets (Fig. 2e) [42, 43]. Moreover, Fourier-transform infrared spectroscopy (FTIR) spectrum of Cu-BDC nanosheets was shown in Fig. 2f. The characteristic peaks at 1578, 1501, 1156, and 1017 cm^−1^ belonging to the benzene rings of the ligands were observed. The characteristic bands at 1624 and 1439 cm^−1^ were indexed to the symmetric and antisymmetric stretching vibrations of –COOH group. Furthermore, thermogravimetric analysis (TGA) was performed to investigate the thermal stability of Cu-BDC nanosheets. As depicted in Fig. 2g, the obtained TGA profile of Cu-BDC nanosheets under air (or N_2_) atmosphere illustrated that the structure of Cu-BDC remained stable up to 300 °C. The weight loss was determined to be 16 wt% by heating to 300 °C, which was attributed to the liberation of the coordinated *N*, *N*-dimethylformamide (DMF) molecule. The occurrence of weight loss was observed ranging from 300 to 330 °C, owing to the decomposition of the BDC ligand of Cu-BDC nanosheets. Furthermore, the Brunauer–Emmett–Teller (BET) surface areas were determined to be 53.2, 254.7, and 307.6 m^2^ g^−1^ for the Cu-BDC nanosheets pretreated at 120, 200, and 250 °C for 12 h before examination, respectively, indicating the porosity evolution after heat treatments by getting rid of the guest DMF molecules.

### In vitro Fenton-like reaction enabled by 2D PEG/Cu-BDC nanocatalysts

**Fig. 3:**
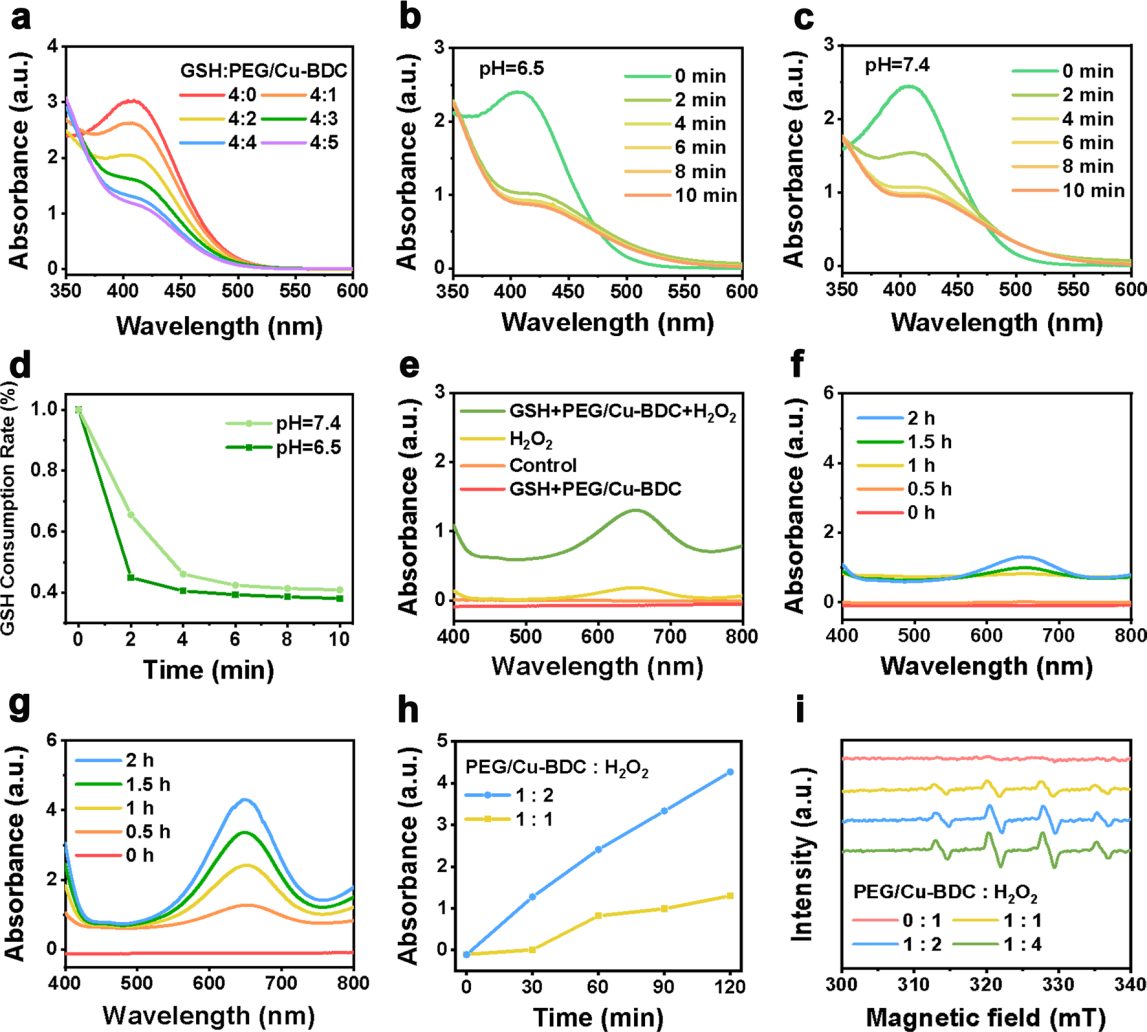
In vitro GSH depletion and ·OH generation enabled by PEG/Cu-BDC. **a** UV–Vis absorption spectra of DTNB solution containing different proportions of GSH and PEG/Cu-BDC. **b**, **c** UV–Vis absorption spectra of DTNB solution containing GSH and PEG/Cu-BDC at (**b**) pH 6.5 and (**c**) pH 7.4. **d** The comparison of GSH consumption rate under pH 6.5 and 7.4, respectively. **e** UV–Vis absorption spectra of oxidized TMB solution after various treatments. **f**, **g** UV–Vis absorption spectra of oxidized TMB solution containing the proportions of PEG/Cu-BDC to H_2_O_2_ of (**f**) 1: 1 and (**g**) 1:2. **h** The ·OH generation rate in different proportions of PEG/Cu-BDC to H_2_O_2_. **i** EPR spectra of DMPO containing different proportions of PEG/Cu-BDC to H_2_O_2_

There were two necessary procedures in the Fenton-like reaction mediated by PEG/Cu-BDC, GSH consumption and ·OH generation. The GSH depletion was confirmed with the assistance of an indicator, DTNB [5,5′-dithio-bis-2-(nitrobenzoic acid)], which generates yellow compound (5-thio-2-nitrobenzoic acid) showing a specific peak at 405 nm after reaction with GSH. As displayed in Fig. 3a, the GSH level kept declining with the elevating concentration of PEG/Cu-BDC. In addition, the GSH depletion was evidently accelerated after incubation of PEG/Cu-BDC at pH 6.5 under the same condition (Fig. 3b–d). The results illustrated that the obtained PEG/Cu-BDC significantly consumed GSH in acidic TME. Furthermore, the ·OH generation property of PEG/Cu-BDC at different pHs was also assessed using a ·OH indicator, 3,3′,5,5′-tetramethylbenzidine (TMB), which exhibits the characteristic peak at 652 nm after oxidation by ·OH. As depicted in Fig. 3e, negligible absorbance change at 652 nm can be detected after incubation of TMB with H_2_O_2_. In contrast, PEG/Cu-BDC accelerated the ·OH generation in the presence of GSH and H_2_O_2_ by measuring the absorbance of TMB at 652 nm, while a slight increment in the absorbance at 652 nm can be observed after incubation of TMB with PEG/Cu-BDC in the presence of GSH. In addition, the ·OH generation enhanced with the extension of reaction durations as well as the increasing proportion of H_2_O_2_ (Fig. 3f–h). Furthermore, the electron spin resonance (ESR) spectroscopy was also recorded to confirm the ·OH production using a ·OH capture agent, 5, 5-dimethyl-1-pyrroline-Noxide (DMPO). The presence of the characteristic signals in the ESR spectra validated the high capability of PEG/Cu-BDC in generating ·OH (Fig. 3i). All these results confirmed that GSH and H_2_O_2_ can serve as an “AND” logic gate to activate the Cu^+^-catalyzed Fenton-like reactions for efficient ·OH generation in the presence of PEG/Cu-BDC nanocatalysts.

### In vitro tumor cell-killing activity of 2D PEG/Cu-BDC nanocatalysts

**Fig. 4:**
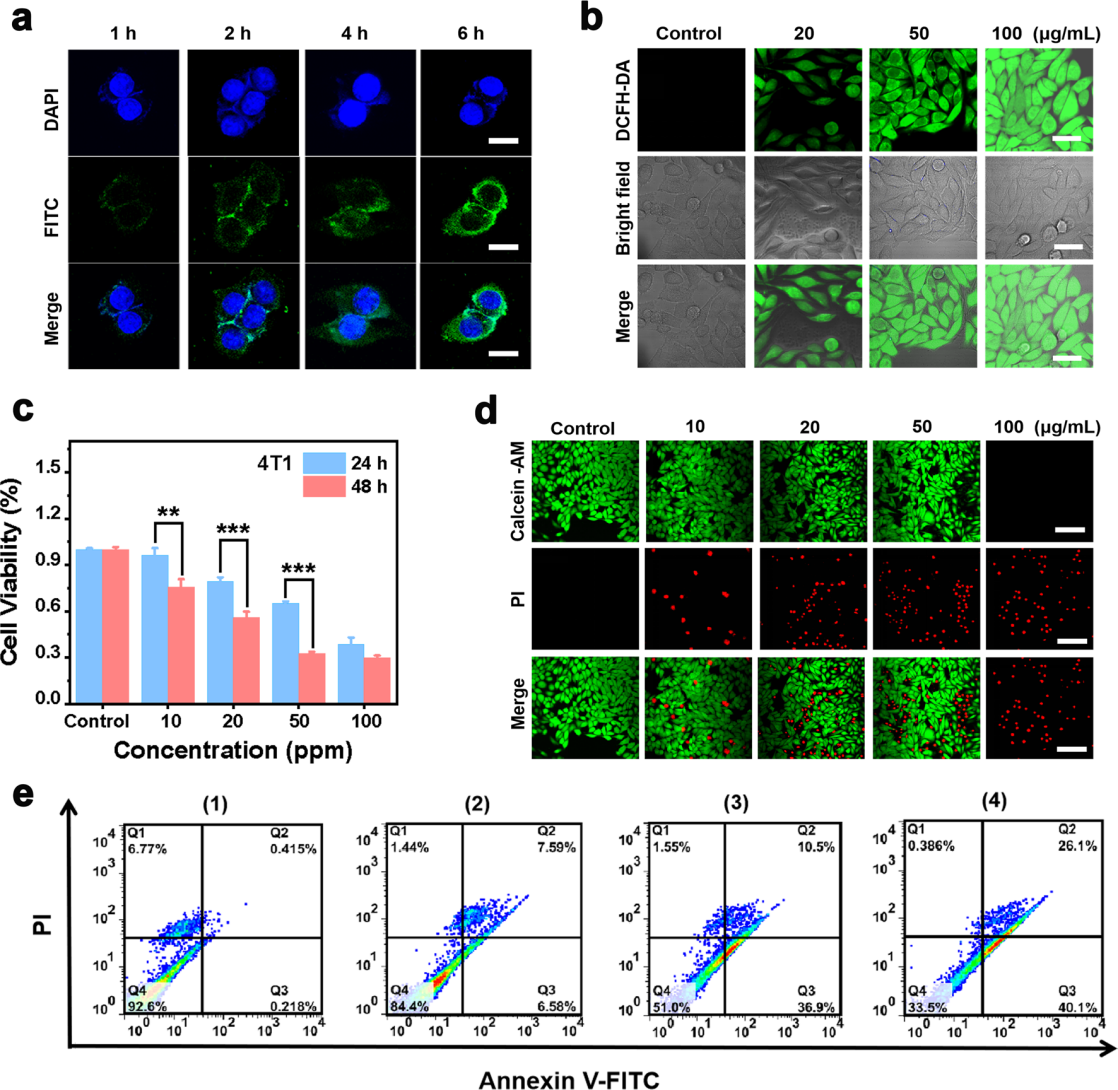
Cellular uptake and therapeutic efficacy of PEG/Cu-BDC against 4T1 breast tumor cells. **a** CLSM images of 4T1 breast tumor cells after incubated with FITC-labeled PEG/Cu-BDC for various durations (1, 2, 4, and 6 h). Scale bars: 30 μm. **b** Intracellular ROS generation in 4T1 breast tumor cells after cultured with various concentrations of PEG/Cu-BDC (0, 20, 50, and 100 μg mL^−1^). Scale bars: 50 μm. **c** Cellular viability of the 4T1 breast tumor cells after incubated with PEG/Cu-BDC for 24 and 48 h, respectively (*n* = 5 biologically independent samples). **d** Live/dead staining of 4T1 breast tumor cells with calcein AM and PI, respectively. Scale bars: 100 μm. **e** Flow cytometry analysis showing apoptosis of 4T1 breast tumor cells after incubation with different concentrations (0, 20, 50, and 100 μg mL^−1^) of PEG/Cu-BDC for 24 h

**Fig. 5:**
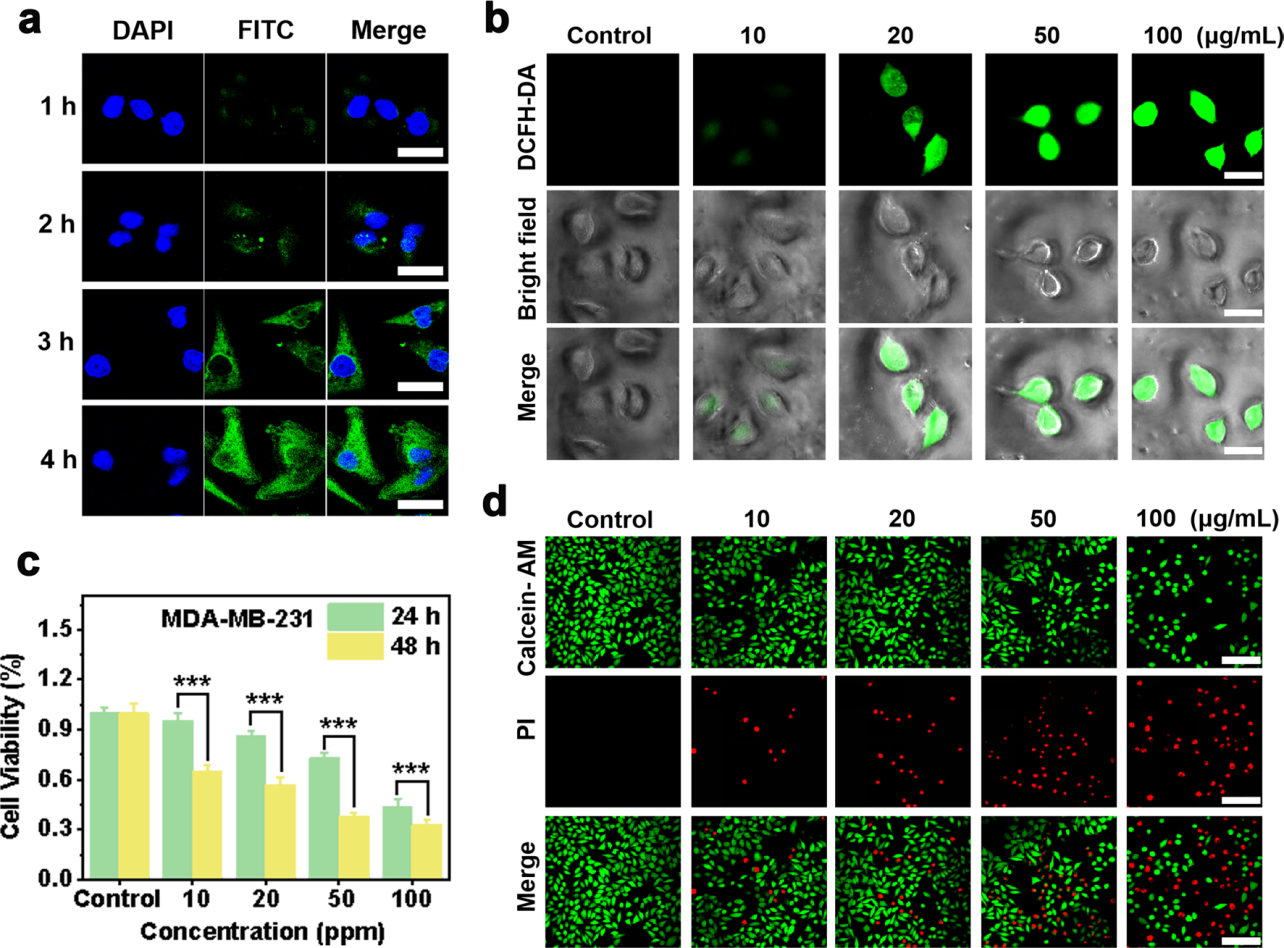
Cellular uptake and therapeutic efficacy of PEG/Cu-BDC against MDA-MB-231 breast tumor cells. **a** CLSM images of MDA-MB-231 breast tumor cells after incubated with FITC-labeled PEG/Cu-BDC for various durations (1, 2, 3, and 4 h). Scale bars: 30 μm. **b** Intracellular ROS generation in MDA-MB-231 breast tumor cells after cultured with various concentrations of PEG/Cu-BDC (0, 10, 20, 50, and 100 μg mL^−1^). Scale bars: 50 μm. **c** Cellular viability of MDA-MB-231 breast tumor cells after incubated with PEG/Cu-BDC for 24 and 48 h, respectively (*n* = 5 biologically independent samples). **d** Live/dead staining of MDA-MB-231 breast tumor cells with calcein AM and PI, respectively. Scale bars: 100 μm

The intracellular uptake of 2D PEG/Cu-BDC nanocatalysts was assessed using confocal laser scanning microscopy (CLSM) observation after incubation of 4T1 and MDA-MB-231 breast tumor cells with fluorescein isothiocyanate (FITC)-labeled PEG/Cu-BDC for different durations. On the basis of the obtained CLSM images, the green fluorescence of FITC-labeled PEG/Cu-BDC enhanced with the extension of the incubation duration, which demonstrated that PEG/Cu-BDC could be efficiently endocytosed by 4T1 and MDA-MB-231 breast tumor cells (Figs. 4a, 5a). After confirmation of the efficient internalization of PEG/Cu-BDC, the ROS generation in 4T1 and MDA-MB-231 breast tumor cells was evaluated after incubation with various concentrations of PEG/Cu-BDC using 2, 7-dichlorodihydrofluorescein diacetate (DCFH-DA) as an indicator. The green fluorescence signal brightened with the increasing concentration of PEG/Cu-BDC, and the signal reached the maximum at an incubation concentration of 100 μg mL^−1^, demonstrating the efficient ROS generation enabled by PEG/Cu-BDC nanocatalysts (Figs. 4b, 5b).

The tumoricidal activity of PEG/Cu-BDC nanocatalysts against 4T1 and MDA-MB-231 breast tumor cells was then assessed by the standard counting kit-8 (CCK-8) assay, CLSM observation, and flow cytometry (FCM) analysis. To further quantitively investigate the anti-tumor efficiency, 4T1 and MDA-MB-231 breast tumor cells were cultured with different concentrations of PEG/Cu-BDC for 24 and 48 h. As depicted in Figs. 4c and 5c, the cell viabilities of 4T1 breast tumor cells were 38.6% and 29.86%, respectively, corresponding to 44.23% and 33.07% for MDA-MB-231 breast tumor cells after treatment with 100 μg mL^−1^ of PEG/Cu-BDC for 24 and 48 h. To confirm the high biocompatibility of PEG/Cu-BDC nanocatalyst towards normal cells, the cell viability of PEG/Cu-BDC nanocatalyst against normal endothelial cells was also evaluated. As shown in Additional file 1: Fig. S1, the cell viability was determined to be 90.3% after treatment with 100 μg mL^−1^ for normal endothelial cells, which was significantly higher than that of 38.6% for 4T1 cells. Additionally, a calcein acetoxymethyl ester (calcein-AM) and propidium iodide (PI) assay was further applied to distinguish the live and dead cells by means of color directly. As presented in Figs. 4d and 5d, the red PI fluorescence signal enhanced with the elevating concentration of PEG/Cu-BDC. Furthermore, the flow cytometry analysis was performed to illustrate that the therapeutic efficiency enhanced with the elevated concentration, which was in accordance with the CLSM observation results (Fig. 4e).

**Fig. 6:**
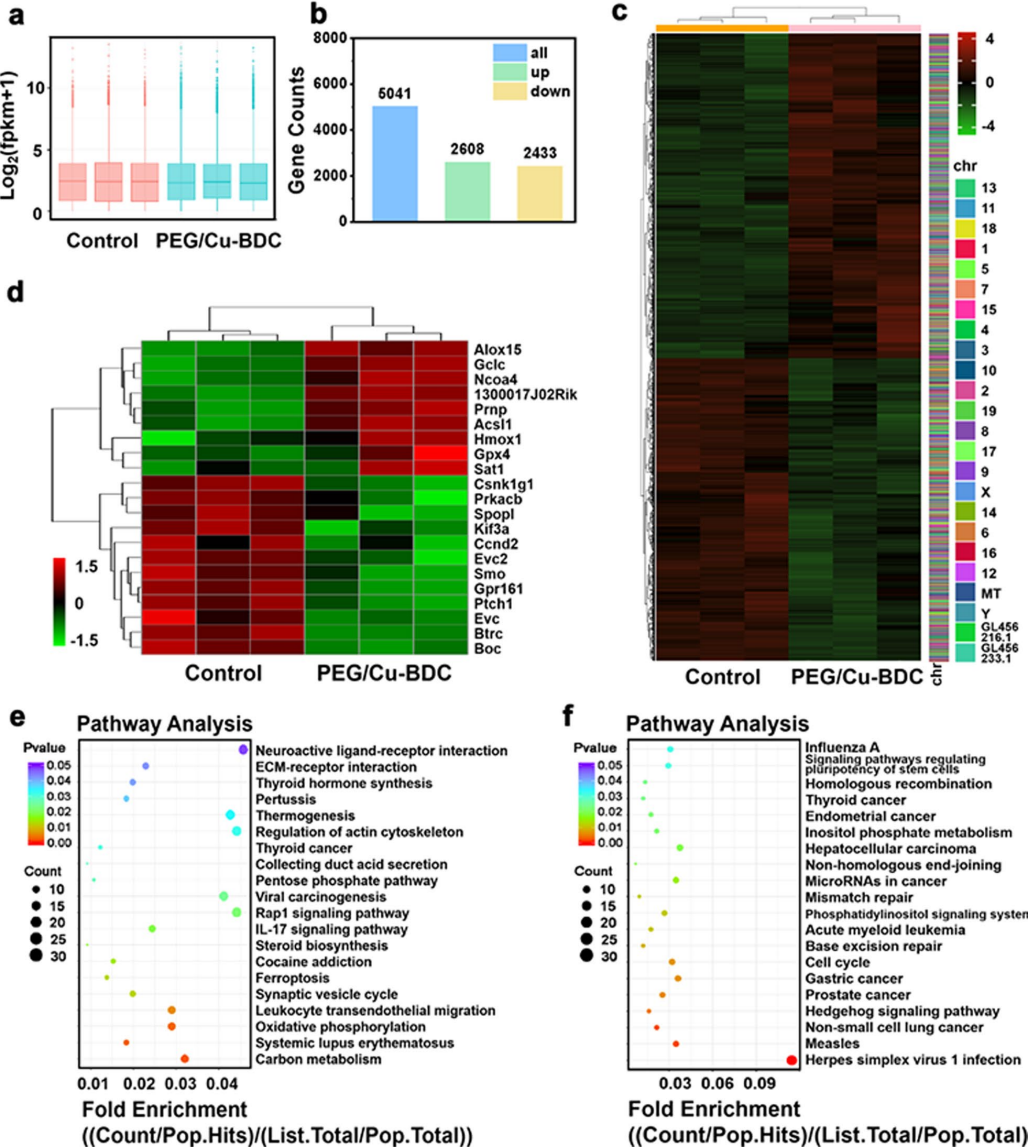
Mechanistic study of 2D PEG/Cu-BDC nanocatalysts in inducing augmented CDT effect. **a** The box plot of these 6 samples, in which all samples were almost at the same level, indicating the quality homogenization of the cell samples. **b** The chart showing the differentially expressed genes in saline- and PEG/Cu-BDC-treated groups (*n* = 3 biologically independent samples). **c** The gene expression heat-map of 4T1 cells in saline- and PEG/Cu-BDC-treated groups (*n* = 3 biologically independent samples). **d** The gene expression heat-map of the related genes in the ferroptosis and Hedgehog signaling pathways. **e**, **f** The top 20 (**e**) up-regulated, and (**f**) down-regulated KEGG pathways after treatment with saline and PEG/Cu-BDC nanosheets (P-value < 0.05)

RNA sequencing was further conducted to investigate the antitumor mechanism of PEG/Cu-BDC by analyzing the mRNA profiling in 4T1 cells after incubation with saline and PEG/Cu-BDC nanosheets, respectively. As shown in Fig. 6a, the box plots in relation with 6 different groups (3 groups for control and 3 groups for PEG/Cu-BDC) were at the same level, which revealed the homogeneity of cell samples, laying foundation for the following mechanism investigation. There were 5041 differentially expressed genes in both saline and PEG/Cu-BDC treated groups, including 2608 up-regulated and 2433 down-regulated ones, respectively (Fig. 6b, c). On the basis of the differentially expressed genes, several key genes that are relevant to cell apoptosis and proliferation were listed in the heat map after treatment with PEG/Cu-BDC incubation. Among these differentially expressed genes associated with ferroptosis [44] and Hedgehog signaling pathway [45], 9 and 12 genes were up-regulated and down-regulated, respectively (Fig. 6d). Furthermore, gene ontology (GO) (Additional file 1: Figs. S2, S3) and Kyoto Encyclopedia of Genes and Genomes (KEGG) (Fig. 6e, f) pathway enrichment analysis were performed to understand how Cu-BDC nanosheets acted on tumor cells by GSH “AND” H_2_O_2_-activated CDT. In the midst of these KEGG pathways, ferroptosis is the main cause in inducing cell death of PEG/Cu-BDC, in which the genetic expression in the glutathione metabolism pathway was up-regulated, thus leading to GSH depletion and ROS elevation in 4T1 cells (Additional file 1: Fig. S4).

### In vivo therapeutic efficacy of 2D PEG/Cu-BDC nanocatalysts

**Fig. 7:**
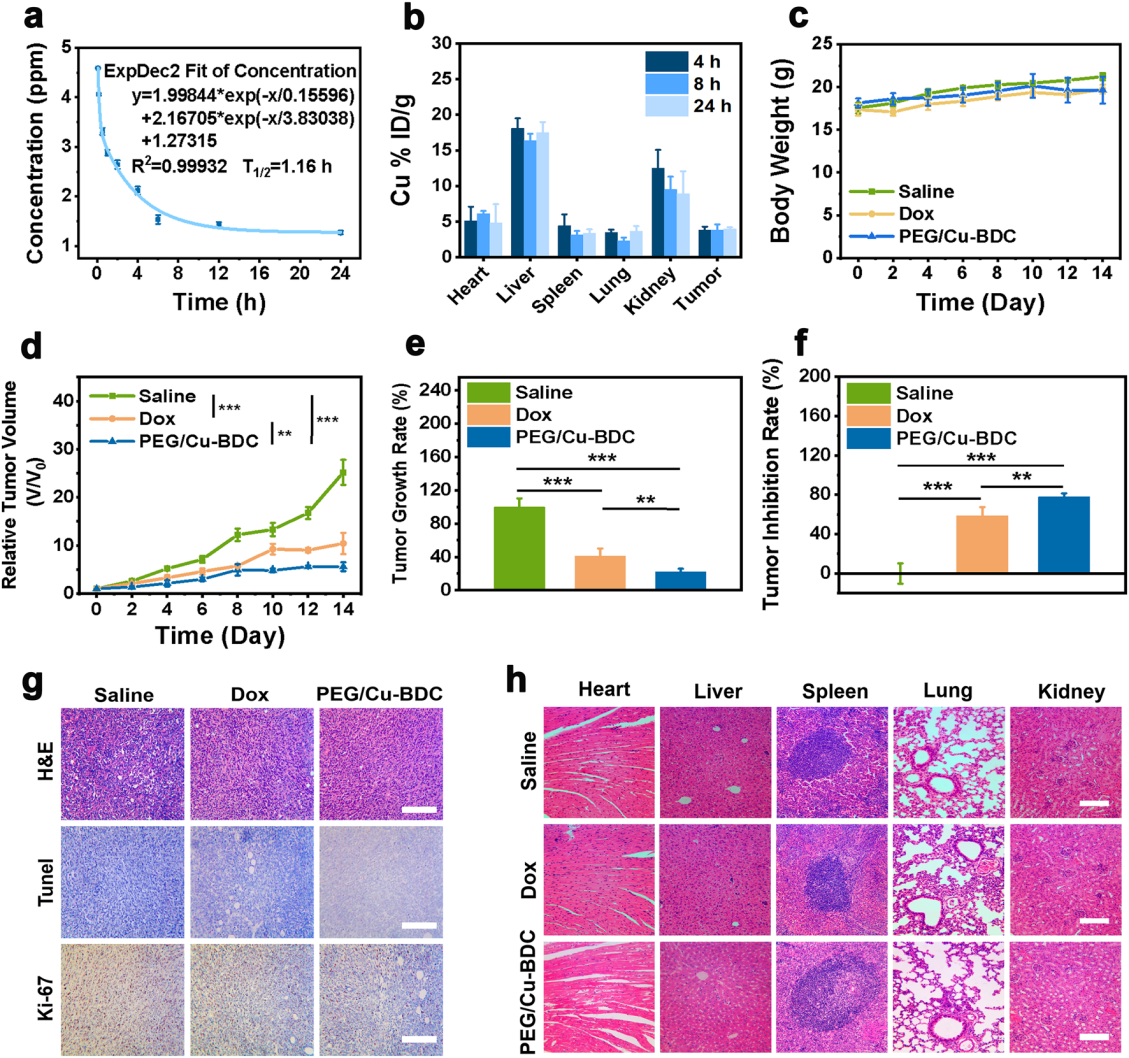
In vivo biological behaviors and therapeutic efficacy of 2D PEG/Cu-BDC nanocatalysts on 4T1 tumor-bearing mice. **a** In vivo pharmacokinetic profile of PEG/Cu-BDC (*n* = 3 biologically independent samples). **b** The biodistribution of PEG/Cu-BDC in tumor tissues and major organs (heart, liver, lung, spleen, and kidney) at different durations post-injection (4, 8, and 24 h) (*n* = 3 biologically independent samples). **c** Body weight variations, **d** relative tumor volumes, **e** tumor growth rates, and **f** tumor inhibition rates of the mice in different treatment groups (P values: *P < 0.05, **P < 0.01, and ***P < 0.001). **g** H&E, TUNEL, and Ki-67 staining of tumor sections (Scale bar: 100 μm), and **h** H&E staining of the major organs in different treatment groups (Scale bar: 100 μm)

Biological behaviors comprehending blood half-life, biodistribution, and biocompatibility were the preconditions of drug action for in vivo application. As depicted in Fig. 7a, the blood half-life of 2D PEG/Cu-BDC nanocatalysts was determined to be 1.16 h after intravenous injection, which revealed the desirable pharmacokinetic performance of PEG/Cu-BDC. In addition, the biodistribution investigation was also performed to reveal the accumulation of PEG/Cu-BDC in tumor tissues and major organs at 4, 8, and 24 h after intravenous administration. As shown in Fig. 7b, PEG/Cu-BDC nanosheets mainly accumulated in the liver tissues, and their accumulation in tumor tissues was determined to be 3.98% at 24 h post-injection.

The systemic toxicity of 2D PEG/Cu-BDC nanocatalysts in vivo was assessed by blood analysis and hematoxylin–eosin (H&E) staining of the major visceral organs of the healthy female Kunming mice. As shown in Additional file 1: Fig. S5, the routine blood parameters and serum biochemical indexes exhibited no significant difference in all treatment groups. In addition, the main organs in both control and PEG/Cu-BDC-treated groups were collected for H&E staining. Negligible inflammation lesions and damage signals were observed for the main organs in all treatment groups, indicating high biocompatibility and biosafety of the engineered PEG/Cu-BDC nanocatalysts for potential therapeutic use (Additional file 1: Figs. S6, S7).

**Fig. 8:**
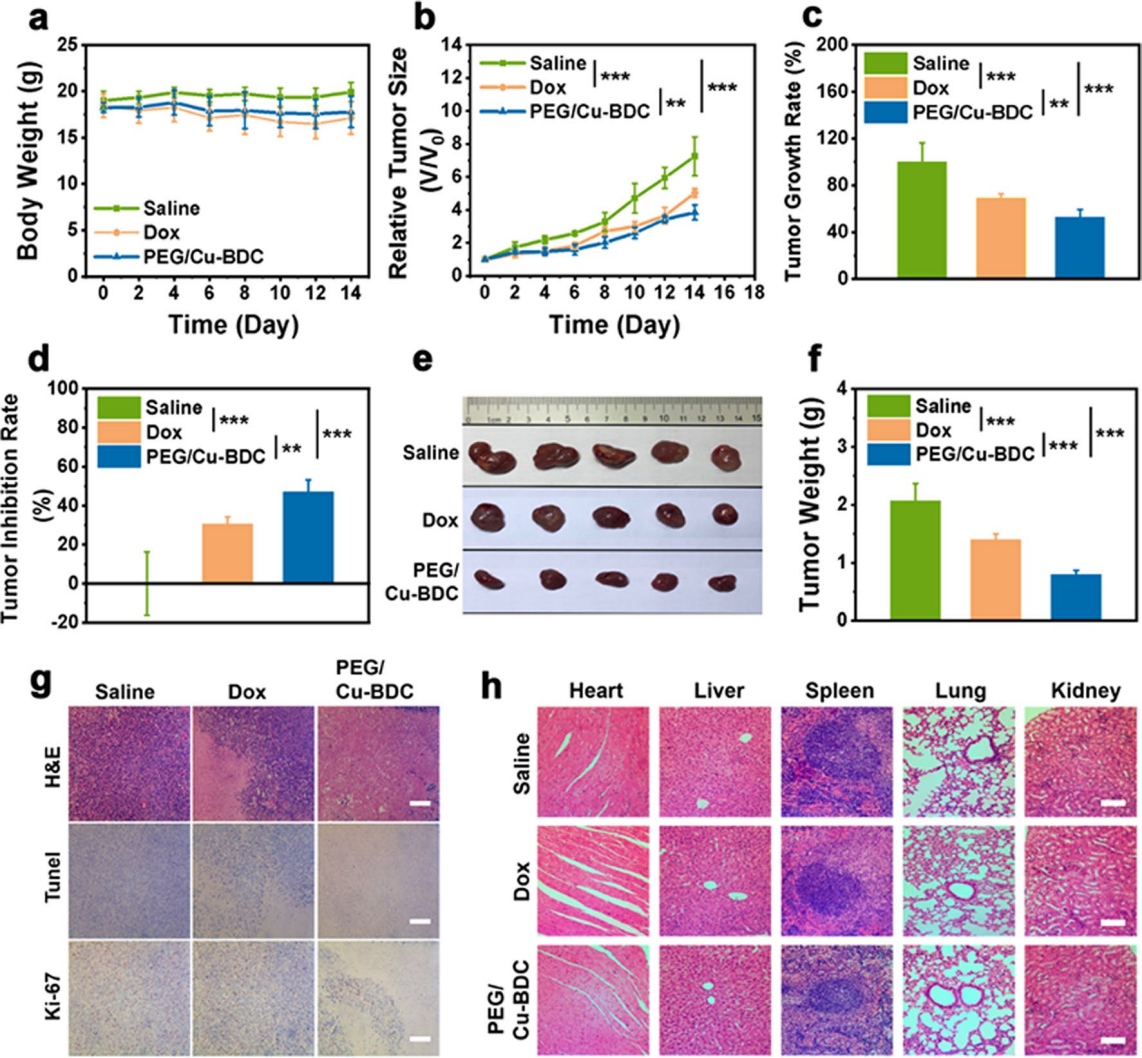
The therapeutic efficacy of 2D PEG/Cu-BDC nanocatalysts on MDA-MB-231 tumor-bearing mice. **a** Body weight variations, **b** relative tumor volumes, **c** tumor growth rates, **d** tumor inhibition rates, and **f** tumor weights of the tumor-bearing mice in different treatment groups (P values: *P < 0.05, **P < 0.01, and ***P < 0.001). **e** Photographs of the tumors dissected from the tumor-bearing mice after various treatments. **g** H&E, TUNEL, and Ki-67 staining of the tumor sections in different treatment groups (Scale bars: 50 μm). **h** H&E staining of the major organs after different treatments (Scale bars: 50 μm)

The antineoplastic activity of the engineered 2D PEG/Cu-BDC nanocatalysts was assessed on female nude mice embedding with 4T1 and MDA-MB-231 breast tumor cells subcutaneously. The mice in three groups were injected with saline, doxorubicin (10 mg kg^−1^), and PEG/Cu-BDC (10 mg kg^−1^), respectively. The body weights and tumor sizes of the mice were recorded. During the therapeutic processes, whether in the 4T1 or MDA-MB-231 breast tumor-bearing mice, the quantitative values and the variation tendency of the body weights in all groups were almost identical, indicating that negligible toxicity in vivo was caused by PEG/Cu-BDC administration (Figs. 7c, 8a). In 4T1 breast tumor-bearing mice, after 14-day treatment, the relative tumor volume (V/V_0_) in the control and doxorubicin-treated groups reached 25.2 and 10.43 with the tumor growth rate of 100% and 41.37%, respectively, while the value of V/V_0_ in the PEG/Cu-BDC-treated group was merely 5.59 with the growth rate of 22.17% (Fig. 7d). In terms of MDA-MB-231 breast tumor-bearing mice, the value of V/V_0_ and tumor growth rate in the PEG/Cu-BDC treatment group approached half of that in the saline-treated group (Fig. 8b, c). Additionally, the average tumor weights, tumor sizes or growth inhibition rates in all treatment groups intuitively validated that severe damage against tumor tissues was caused by PEG/Cu-BDC administration (Figs. 7e, f, 8d–f).

Furthermore, the histological evaluation of representative tumor tissues of the mice in all treatment groups was conducted to investigate the therapeutic mechanism of PEG/Cu-BDC through H&E and terminal deoxynucleotidyl transferase uridine triphosphate nick end labeling (TUNEL) staining. In comparison with doxorubicin-treated group, more prominent histological damage signal was observed in the PEG/Cu-BDC-treated group from H&E and TUNEL staining images (Figs. 7g, 8g). Additionally, Ki-67 antibody staining assay was performed to assess the tumor-cell proliferative property of the mice in various treatment groups. By comparing the Ki-67 staining images in other treatment groups, PEG/Cu-BDC presented conspicuous suppressive effect on the proliferative activity of tumor tissues. To better validate the therapeutic efficacy of PEG/Cu-BDC nanocatalyst, the quantitative analysis of Ki-67 and terminal deoxynucleotidyl transferase dUTP nick-end labeling (TUNEL) staining was performed. In comparison with the control group, the percentages of Ki-67 positive tumor cells were distinctly decreased to 23.3% and 12.5% for PEG/Cu-BDC nanocatalyst-treated 4T1 and MDA-MB-231 tumor-bearing mice, respectively, while the numbers of apoptotic cells were apparently elevated (80.7% and 66.7% for 4T1 and MDA-MB-231 tumor-bearing mice, respectively) after treatment with PEG/Cu-BDC nanocatalyst (Additional file 1: Figs. S8, S9). Moreover, no obvious damage signal of the main organs was detected from H&E staining images in different treatment groups, demonstrating that PEG/Cu-BDC features negligible adverse effect on the health of the mice (Figs. 7h, 8h).

## Conclusions

In summary, we have engineered a distinct 2D Cu (II)-based MOF nanosheets as an iron-free nanocatalyst for GSH-activated and H_2_O_2_-reinfored chemodynamic tumor therapy via sequential reaction with intracellular GSH and H_2_O_2_ to induce ·OH generation. The in vitro and in vivo assessments verified that the achieved Cu-BDC nanosheets efficiently induced cellular apoptosis and promoted tumor inhibition in situ without apparent systematic toxicity, compared with the inferior tumor suppression and high systemic toxicity of the equivalent concentration of commercial chemotherapeutic drug, Dox. Therefore, PEG/Cu-BDC exhibits high potential in the application of non-ferrous high-performance Fenton catalyst for tumor CDT treatment as well as broadens the application domain in MOF-based nanomaterials in disease diagnosis and treatment.

## Supplementary Information

**Additional file 1**: **Figure S1. **Cell viability of 4T1 and normal endothelial cells after incubated with various concentrations of PEG/Cu-BDC for 24 h, respectively. **Figures S2.** The GO enriched pathways ranked top ten in terms of credibility in biological process. (P-value < 0.05). **Figures S3.** The GO descending pathways ranked top ten in terms of credibility in biological process. (P-value < 0.05). **Figures S4.** Illustration of ferroptosis signaling pathway. The red blocks represent up-regulated genes. **Figure S5. **Routine blood parameters and serum biochemical indexes of female Kunming mice after intravenous injection with 10 or 20 mg kg^−1^ of PEG/Cu-BDC at the 0, 3rd, 7th, 15th, and 30th day, respectively (red and yellow for 10 mg kg^−1^, blue and green for 20 mg kg^−1^). **Figure S6. **H&E staining images of major organs (heart, liver, spleen, lung, and kidney) from female Kunming mice after injection with 10 mg kg^−1^ of PEG/Cu-BDC at the 0, 3rd, 7th, 15th, and 30th day (Scale bar: 100 μm). **Figure S7. **H&E staining of major organs (heart, liver, spleen, lung, and kidney) from female Kunming mice after intravenous injection with 20 mg kg^−1^ of PEG/Cu-BDC at the 0, 3rd, 7th, 15th, and 30th day (Scale bar: 100 μm). **Figure S8. **Quantitative analysis of Ki-67 and apoptosis-positive tumor cells of 4T1 tumor-bearing mice in different treatment groups. **Figure S9. **Quantitative analysis of Ki-67 and apoptosis-positive tumor cells of MDA-MB-231 tumor-bearing mice in different treatment groups.

## Data Availability

All data generated or analyzed during this study are included in the manuscript and supporting information.
