# Supplementary material for: Engineering 2D Cu-composed metal–organic framework nanosheets for augmented nanocatalytic tumor therapy
Source: J Nanobiotechnology. 2022 Feb 4;20:66. doi: 10.1186/s12951-022-01250-x (PMC8815149; doi:10.1186/s12951-022-01250-x)
Supplement: Supplementary file 1 — Additional file 1: Figure S1. Cell viability of 4T1 and normal endothelial cells after incubated with various concentrations of PEG/Cu-BDC for 24 h, respectively. Figures S2. The GO enriched pathways ranked top ten in terms of credibility in biological process. (P-value < 0.05). Figures S3. The GO descending pathways ranked top ten in terms of credibility in biological process. (P-value < 0.05). Figures S4. Illustration of ferroptosis signaling pathway. The red blocks represent up-regulated genes. Figure S5. Routine blood parameters and serum biochemical indexes of female Kunming mice after intravenous injection with 10 or 20 mg kg−1 of PEG/Cu-BDC at the 0, 3rd, 7th, 15th, and 30th day, respectively (red and yellow for 10 mg kg−1, blue and green for 20 mg kg−1). Figure S6. H&E staining images of major organs (heart, liver, spleen, lung, and kidney) from female Kunming mice after injection with 10 mg kg−1 of PEG/Cu-BDC at the 0, 3rd, 7th, 15th, and 30th day (Scale bar: 100 μm). Figure S7. H&E staining of major organs (heart, liver, spleen, lung, and kidney) from female Kunming mice after intravenous injection with 20 mg kg−1 of PEG/Cu-BDC at the 0, 3rd, 7th, 15th, and 30th day (Scale bar: 100 μm). Figure S8. Quantitative analysis of Ki-67 and apoptosis-positive tumor cells of 4T1 tumor-bearing mice in different treatment groups. Figure S9. Quantitative analysis of Ki-67 and apoptosis-positive tumor cells of MDA-MB-231 tumor-bearing mice in different treatment groups. [file 12951_2022_1250_MOESM1_ESM.docx]

**Supporting information for**

**Engineering 2D Cu-Composed Metal-Organic Framework Nanosheets for Augmented Nanocatalytic Tumor Therapy**

Shangwen Zhuang,^1^ Huijing Xiang,^2^* Yixin Chen,^1^ Lulu Wang,^1^ Yu Chen,^2^*and Jun Zhang^1^*

^1^Department of Radiology, Huashan Hospital, State Key Laboratory of Medical Neurobiology,

Fudan University, Shanghai 200040, P. R. China. Email: [zhangjun_zj@fudan.edu.cn](mailto:zhangjun_zj@fudan.edu.cn).

^2^Materdicine Lab, School of Life Sciences, Shanghai University, Shanghai 200444, P. R. China.

Email: xianghuijing@shu.edu.cn; [chenyuedu@shu.edu.cn](mailto:chenyuedu@shu.edu.cn).

**Materials and Methods**

**Materials**

Copper (II) chloride dihydrate (CuCl_2_·2H_2_O), polyvinylpyrrolidone (PVP; K-30), L-ascorbic acid, sodium hydroxide, ethanol, 1,4-benzenedicarboxylic acid (H_2_BDC), *N*, *N*′-dimethylformamide (DMF), and methanol were obtained from Sigma-Aldrich.

**Characterization**

Transmission electron microscopy (TEM) images were acquired on a JEM-2100F electron microscope at an acceleration voltage of 200 kV. UV-vis-NIR absorption spectra were conducted on a UV-3101PC Shimadzu spectrometer. X-ray diffraction (XRD) patterns were obtained on a Rigaku D/MAX-2200 PX XRD system. The speciﬁc surface area and pore size of nanocomposites were determined by a nitrogen adsorption technique at 77 K on a Micrometitics Tristar 3000 system. X-ray photoelectron spectroscopy (XPS) was conducted on an ESCAlab250 instrument from Thermal Scientiﬁc. Thermogravimetric analysis (TGA, Q50, TA Instruments) was conducted at an elevation rate of 10 °C min^−1^. Fourier transformed infrared (FTIR) spectra were recorded with a Shimadzu IR Prestige-21 FTIR spectrometer. The Confocal laser scanning microscopy (CLSM) images were acquired on an FV1000 (Olympus Company, Japan). Flow cytometry analysis were conducted by BD LSRFortessa. The quantitative analysis was performed by inductively coupled plasma optical emission spectrometer (ICP-OES) (Agilent Technologies, US). All other reagents used were of analytical grade without further purification. Deionized water was obtained from a Millipore water purification system. All the experiments and procedures were performed under guidelines approved by the Institutional Animal Care and Use Committee at Fudan University.

**Synthesis of Cu_2_O Nanocubes**

First, the preparation for 0.2 M NaOH solution and 0.1 M ascorbic acid solution needed to be conducted before the synthesis. After the preparation, 17.1 mg of CuCl_2_ and 0.1 g of PVP were dissolved in 40 mL of water. 2.5 mL of NaOH solution (0.2 M) was dropwise added into the above solution with each drop nearly 30 μL s^-1^. Then, the mixture was magnetically stirred for 5 min, followed by the addition of ascorbic acid solution (2.5 mL, 0.1 M) with a speed of 10 µL s^−1^. The mixed solution was stirred for another 5 min. The product was then washed with ethanol twice and acquired through centrifugation. Finally, the yellow Cu_2_O product were uniformly dispersed into 10 mL of ethanol for further use.

**Synthesis of PEG/Cu-BDC**

0.5 mmol H_2_BDC, 5 mL of ethanol and 5 mL of DMF were magnetically stirred till the mixture was fully dissolved. Then, the already prepared Cu_2_O solution in ethanol was poured into the mixture. The formation of Cu-BDC was allowed at a normal ambient temperature for 4 h in the rich oxygen environment, where the oxygen is sufficient enough to oxidize the released Cu^+^ ions from the Cu_2_O to form the blue product. The Cu-BDC nanosheets were acquired through centrifugation at 11000 rpm for 10 min before washing by methanol. Then, the Cu-BDC nanosheets were sonicated and the resultant dispersion was centrifuged for 5 min at 2000 rpm to remove sediment. The resultant dispersion of Cu-BDC nanosheets was obtained for further use. Finally, the final product was dispersed in 10 mL of methanol containing PEG under magnetic stirring for 8 h for further use.

**Detection of GSH Depletion and •OH** **Generation**

The same concentration of reduced glutathione (GSH) and PEG/Cu-BDC in different ratios (GSH : PEG/Cu-BDC = 4 : 0, 4 : 1, 4 : 2, 4 : 3, 4 : 4) was added into a transparent QS-grade quartz cuvette at room temperature for 10 min. Then, 5, 5’-dithiobis-(2-nitrobenzoic acid) (DTNB, 10 µL, 10 mg mL^-1^) was added into the above solution, and the GSH depletion was detected by UV-vis spectroscopy. In addition, the measurement procedure of hydroxyl radical (•OH) was almost the same as that of GSH except for the addition of 3, 3’, 5, 5’-tetramethylbenzidine dihydrochloride ([TMB•2HCl](https://www.chembk.com/en/chem/TMB·2HCl)) solution and hydrogen peroxide (H_2_O_2_).

**Cell Culture.**

4T1 and MDA-MB-231 breast tumor cells were cultivated in Dulbecco’s Modified Eagle’s Medium (DMEM) mixed with 10% fetal bovine serum (FBS) and 1% streptomycin/penicillin in an atmosphere of 5% carbon dioxide (CO_2_) at 37 °C.

**Cell Uptake of PEG/Cu-BDC *In Vitro***

1 mg of fluorescein isothiocyanate (FITC) in 4 mL of ethanol was added into 1 mL of PEG/Cu-BDC solution (1 mg mL^-1^), and magnetically stirred for 12 h in the darkness. The solution was centrifuged at 11000 rpm for 10 min, and then washed with deionized water. The obtained FITC-labeled PEG/Cu-BDC was dispersed into deionized water for further use.

The cellular uptake of PEG/Cu-BDC was evaluated through confocal laser scanning microscopy (CLSM) observation. 4T1 and MDA-MB-231 tumor cells were seeded for 24 h and incubated with FITC-labeled PEG/Cu-BDC (100 μg mL^-1^) for different durations. Afterwards, 1 mL of 4', 6-diamidino-2-phenylindole (DAPI) was added into the dishes to stain the cell nuclei for 10 min. After washing by PBS for three times, the 4T1 and MDA-MB-231 cells were observed by CLSM.

**Therapeutic Efficacy of PEG/Cu-BDC *In Vitro***

The tumoricidal activity of PEG/Cu-BDC against 4T1 and MDA-MB-231 breast tumor cells was assessed by a standard cell counting kit-8 (CCK-8) assay, CLSM observation, and flow cytometry (FCM) analysis. 4T1 and MDA-MB-231 breast tumor cells were seeded in 96-well plates at a density of 1 × 10^4^ per well overnight and cultured with various concentrations of PEG/Cu-BDC (0, 10, 20, 50 and 100 μg mL^-1^) for 24 and 48 h, respectively. Microplate reader (Bio-Tek ELx800, USA) was applied to assess the cell viability using a CCK-8 assay.

For CLSM observation, 4T1 and MDA-MB-231 tumor cells were seeded into 35 mm glass dishes for 24 h. Then, the cells were incubated with different concentrations of PEG/Cu-BDC (0, 10, 20, 50 and 100 μg mL^-1^) for 24 h. After stained by calcein-AM and PI, the cells were imaged by CLSM to distinguish the live and dead cells.

**Cell Apoptosis of PEG/Cu-BDC**

4T1 cells were seeded into 6-well plates for 24 h, and further incubated with different concentrations of PEG/Cu-BDC (0, 20, 50 and 100 μg mL^-1^) for 24 h. After washing with PBS for three times, the cells were collected by trypsinization, followed by centrifugation at 1000 rpm for 5 min. The obtained precipitate was dispersed in PBS and stained with Annexin V-FITC and PI before performing FCM analysis.

**Intracellular Reactive Oxygen Species (ROS) Generation *In Vitro***

4T1 and MDA-MB-231 breast tumor cells were cultured in 35 mm confocal dishes for 24 h. Then, the cells were cultivated with PEG/Cu-BDC (0, 10, 20, 50 and 100 μg mL^-1^) for 8 h, and stained by 2’, 7’-dichlorodihydrofluorescein diacetate (DCFH-DA) for 15 min. Finally, the cells were washed by PBS for three times and images by CLSM.

**mRNAs Analysis**

RNAs extracted from 4T1 cells in 6 groups, including 3 control and 3 PEG/Cu-BDC treated groups were utilized for gene analysis. Before the utilization of the cBot Cluster Generation System with TruSeq PE Cluster Kit v3-cBot-HS (Illumia) for cluster generation, the RNA integrity should be performed with the RNA Nano 6000 Assay Kit of the Bioanalyzer 2100 system (Agilent Technologies, CA, USA). Those sequences of samples would be illustrated by an Illumina Novaseq platform, and the final gene analysis was performed with the help of the DESeq2 R package (1.20.0). The Gene Ontology (GO) and Kyoto Encyclopedia of Genes and Genomes (KEGG) enrichment pathways were analysed by the clusterProfiler R package. P-value of < 0.05 was considered as a sign of significant change.

**Biocompatibility of PEG/Cu-BDC *In Vivo***

The healthy female Kunming mice were divided into 2 groups, and administrated intravenously with 100 μL of PEG/Cu-BDC at different concentrations (10 and 20 mg kg^-1^). The blood and main organs (heart, liver, spleen, lung, and kidney) were harvested at 0, 3^rd^, 7^th^, 15^th^, and 30^th^ day after intravenous injection for routine blood analysis, serum biochemical examination, and haematoxylin-eosin (H&E) staining.

**Pharmacokinetic Behavior of PEG/Cu-BDC *In Vivo***

PEG/Cu-BDC (200 μL, 0.20 mg mL^−1^) was intravenously injected into female Kunming mice. The blood (10 μL) of the mice was collected at a given time point (5, 10, 30 min, 1, 2, 4, 6, 12, and 24 h) after intravenous administration. The Cu contents in the blood samples were detected by ICP-OES analysis to evaluate the *in vivo* pharmacokinetic behavior of PEG/Cu-BDC.

**Biodistribution of PEG/Cu-BDC *In Vivo***

PEG/Cu-BDC (200 μL, 0.2 mg mL^−1^) was intravenously injected into 4T1 tumor-bearing mice. The tumors and main organs (heart, liver, lung, spleen, and kidney) of the mice were collected at various time durations (4, 8, and 24 h). The Cu content in the tumors and major organs were detected by ICP-OES analysis to evaluate the biodistribution of PEG/Cu-BDC.

**Antitumor Efficacy of PEG/Cu-BDC *In Vivo***

4T1 and MDA-MB-231 breast tumor cells were injected subcutaneously in the right back of female nude mice to establish tumor models for further investigation. When the tumor volume was approximately 100 mm^3^, the mice were randomly divided into three groups (n = 5 in each group): Group 1: saline (100 μL/tumor), Group 2: doxorubicin (10 mg kg^-1^, 100 μL/tumor), and Group 3: PEG/Cu-BDC (10 mg kg^-1^, 100 μL/tumor). Then, the body weight and tumor size were recorded every two days. The mice were sacrificed at the end of the therapeutic process. The representative tumors of the mice were dissected for H&E, TdT-mediated dUTP nick-end labeling (TUNEL), and Ki-67 antibody staining. In addition, the representative major organs (heart, liver, lung, spleen, and kidney) of the mice in all treatment groups were collected for H&E staining.

The volumes of the tumors were calculated by this formula: V=ab^2^/2 (a for length and b for width)

The tumor inhibition rates (TIR) were calculated according to the following formula:

$TIR=(1-\frac{{RVT}_{experiment}}{{RVT}_{control}}$) × 100%

**Statistical analysis**

All data were exhibited as mean ± standard deviation and the statistical comparisons were analyzed through Student’s two-sided t-test as *P < 0.05, **P < 0.01, and ***P < 0.001.

**Supplementary figures**


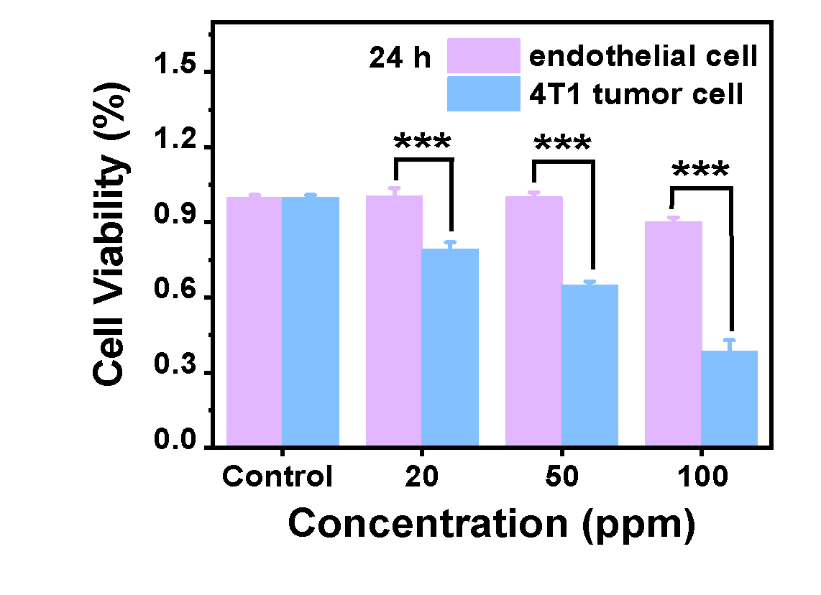


**Figure S1.** Cell viability of 4T1 and normal endothelial cells after incubated with various concentrations of PEG/Cu-BDC for 24 h, respectively.

**
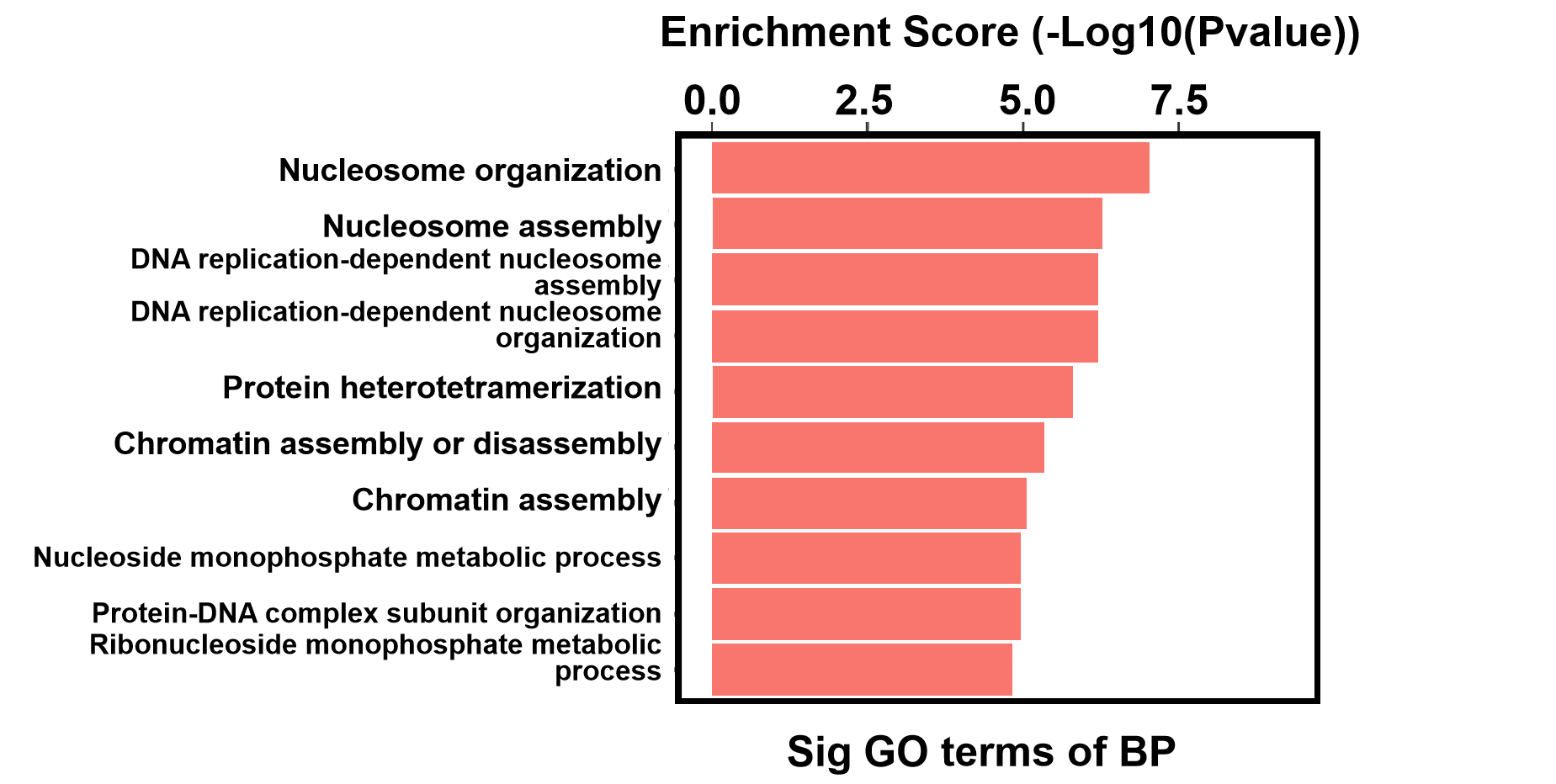
**

**Figures S2.** The GO enriched pathways ranked top ten in terms of credibility in biological process. (P-value < 0.05)


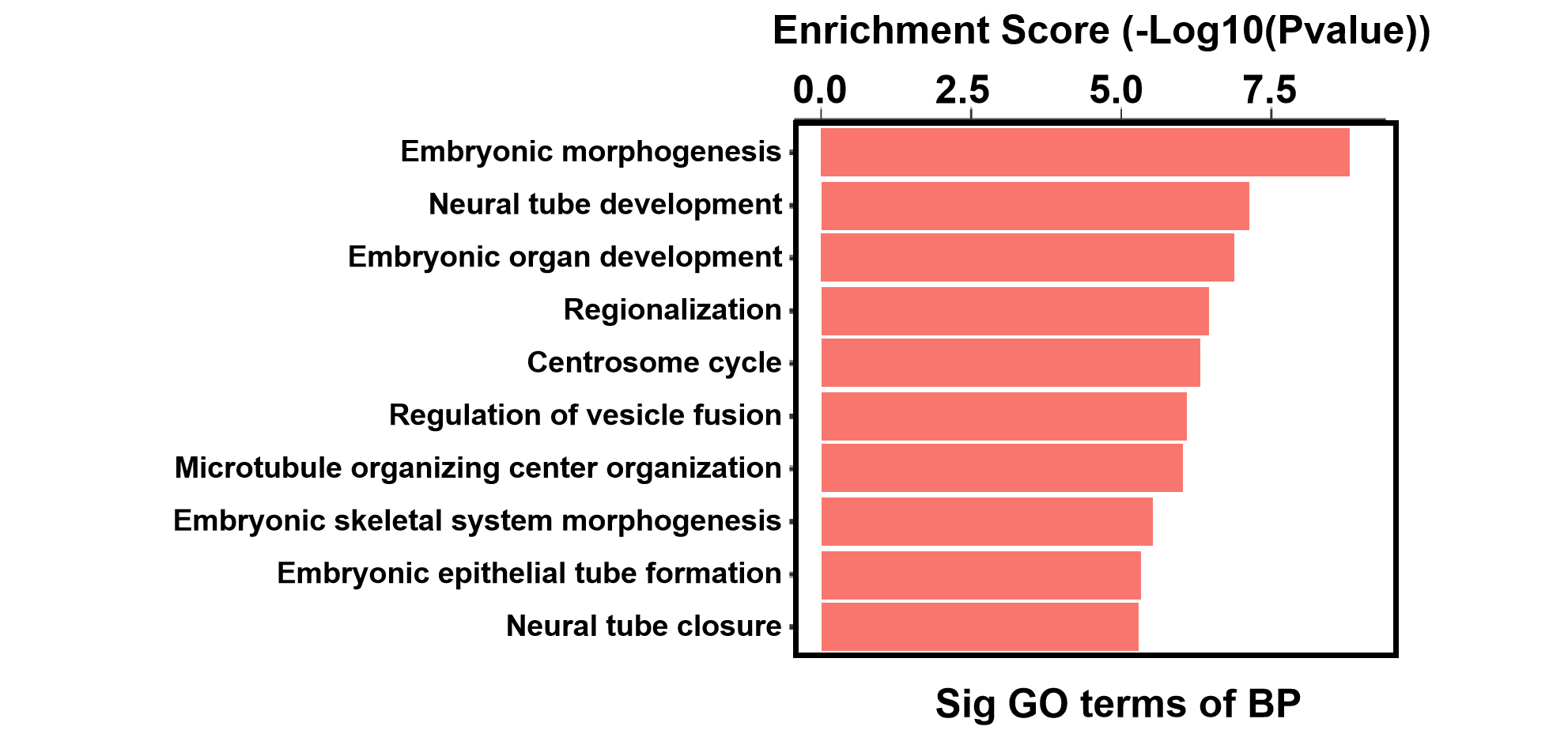


**Figures S3.** The GO descending pathways ranked top ten in terms of credibility in biological process. (P-value < 0.05)


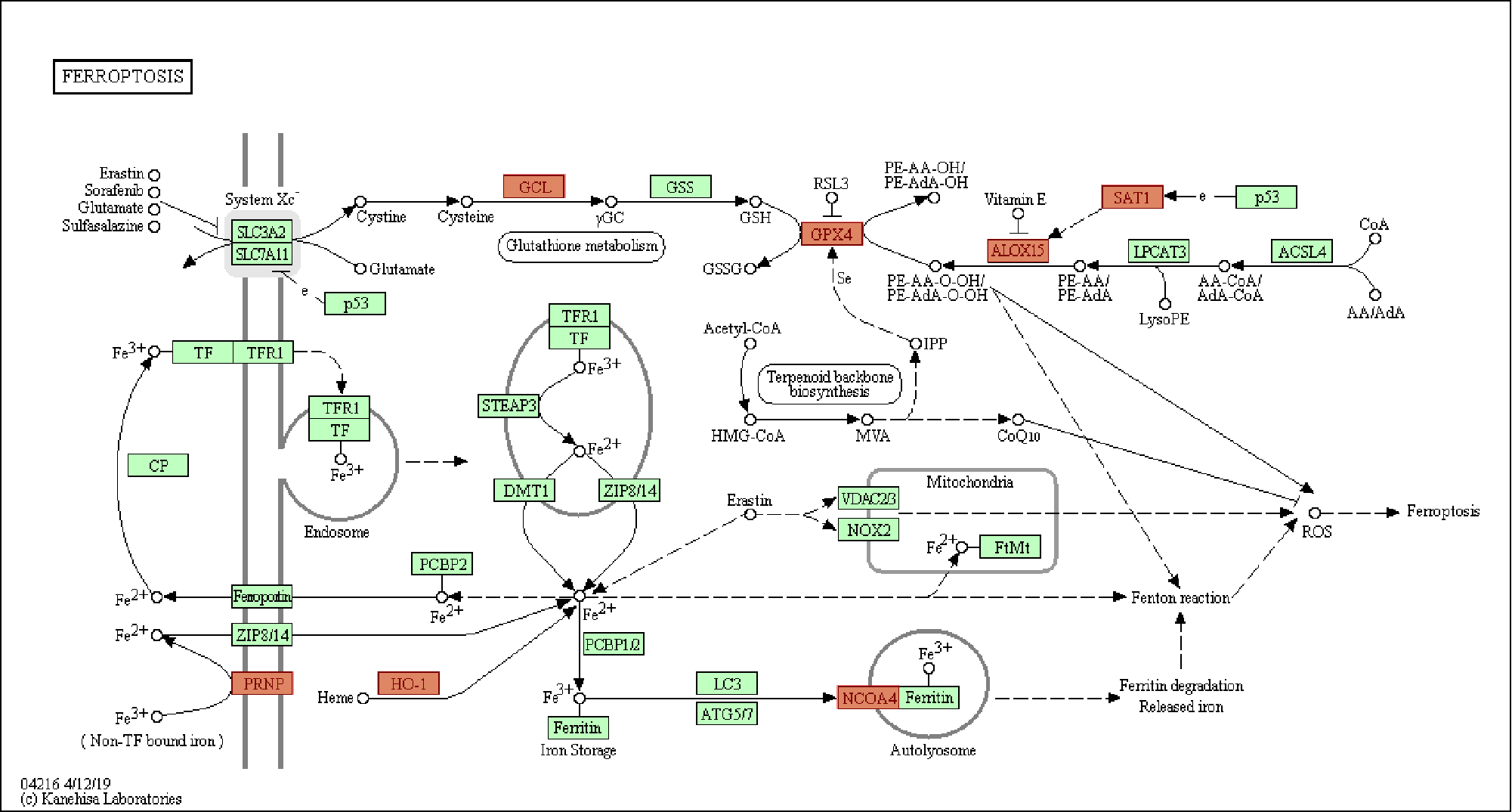


**Figures S4.** Illustration of ferroptosis signaling pathway. The red blocks represent up-regulated genes.

**

**

**Figure S5.** Routine blood parameters and serum biochemical indexes of female Kunming mice after intravenous injection with 10 or 20 mg kg^-1^ of PEG/Cu-BDC at the 0, 3^rd^, 7^th^, 15^th^, and 30^th^ day, respectively (red and yellow for 10 mg kg^-1^, blue and green for 20 mg kg^-1^).


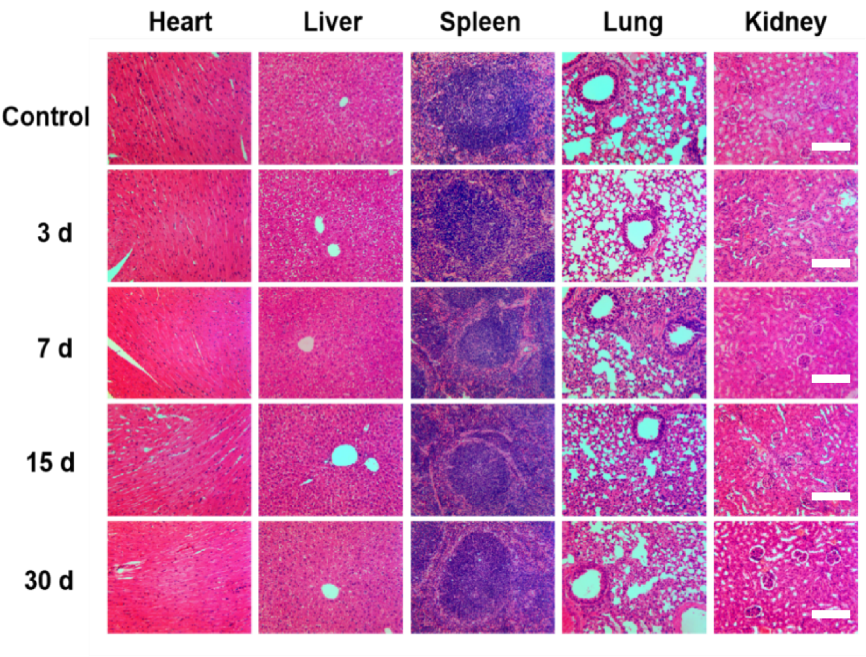


**Figure S6.** H&E staining images of major organs (heart, liver, spleen, lung, and kidney) from female Kunming mice after injection with 10 mg kg^-1^ of PEG/Cu-BDC at the 0, 3^rd^, 7^th^, 15^th^, and 30^th^ day (Scale bar: 100 μm).

**
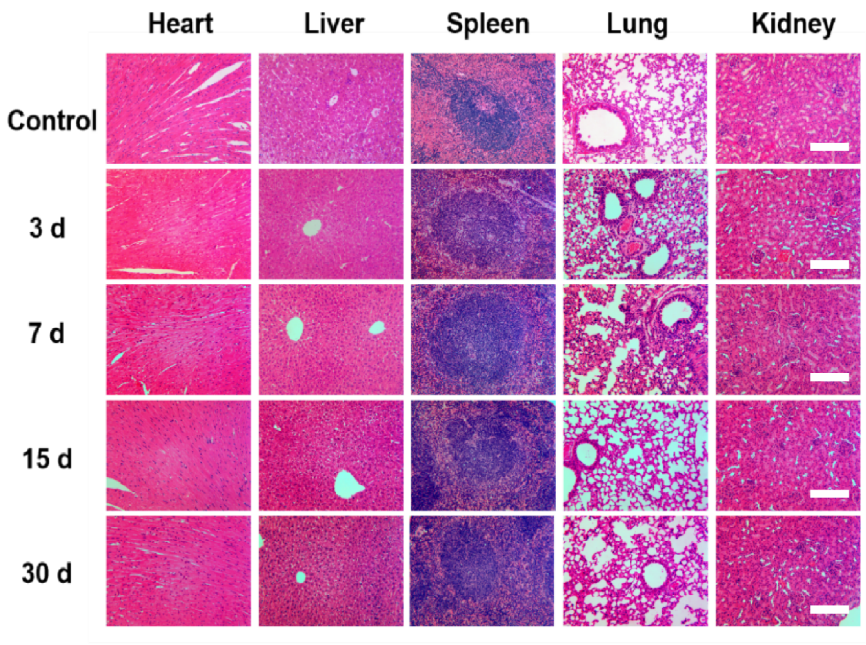
**

**Figure S7.** H&E staining of major organs (heart, liver, spleen, lung, and kidney) from female Kunming mice after intravenous injection with 20 mg kg^-1^ of PEG/Cu-BDC at the 0, 3^rd^, 7^th^, 15^th^, and 30^th^ day (Scale bar: 100 μm).


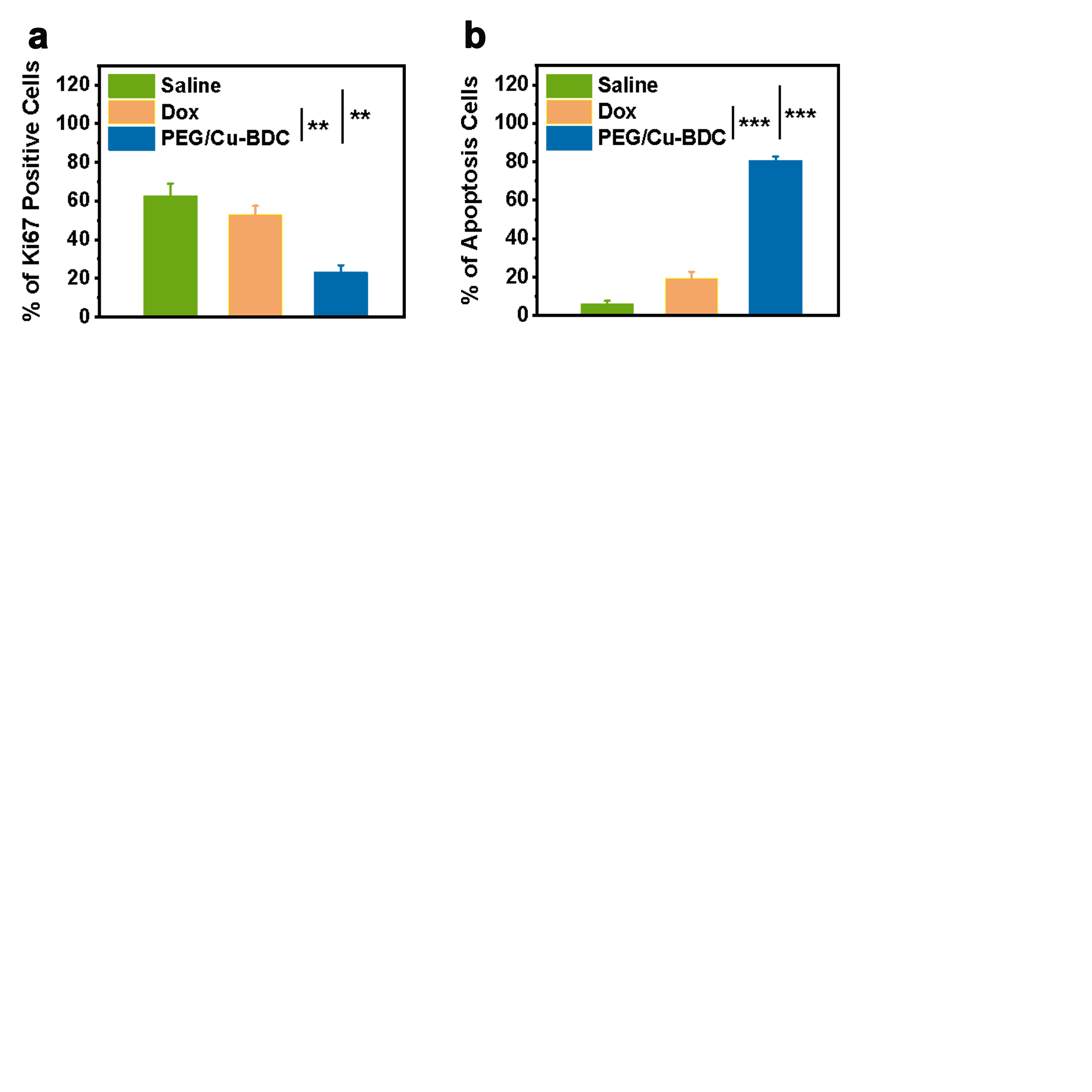


**Figure S8.** Quantitative analysis of Ki-67 and apoptosis-positive tumor cells of 4T1 tumor-bearing mice in different treatment groups.


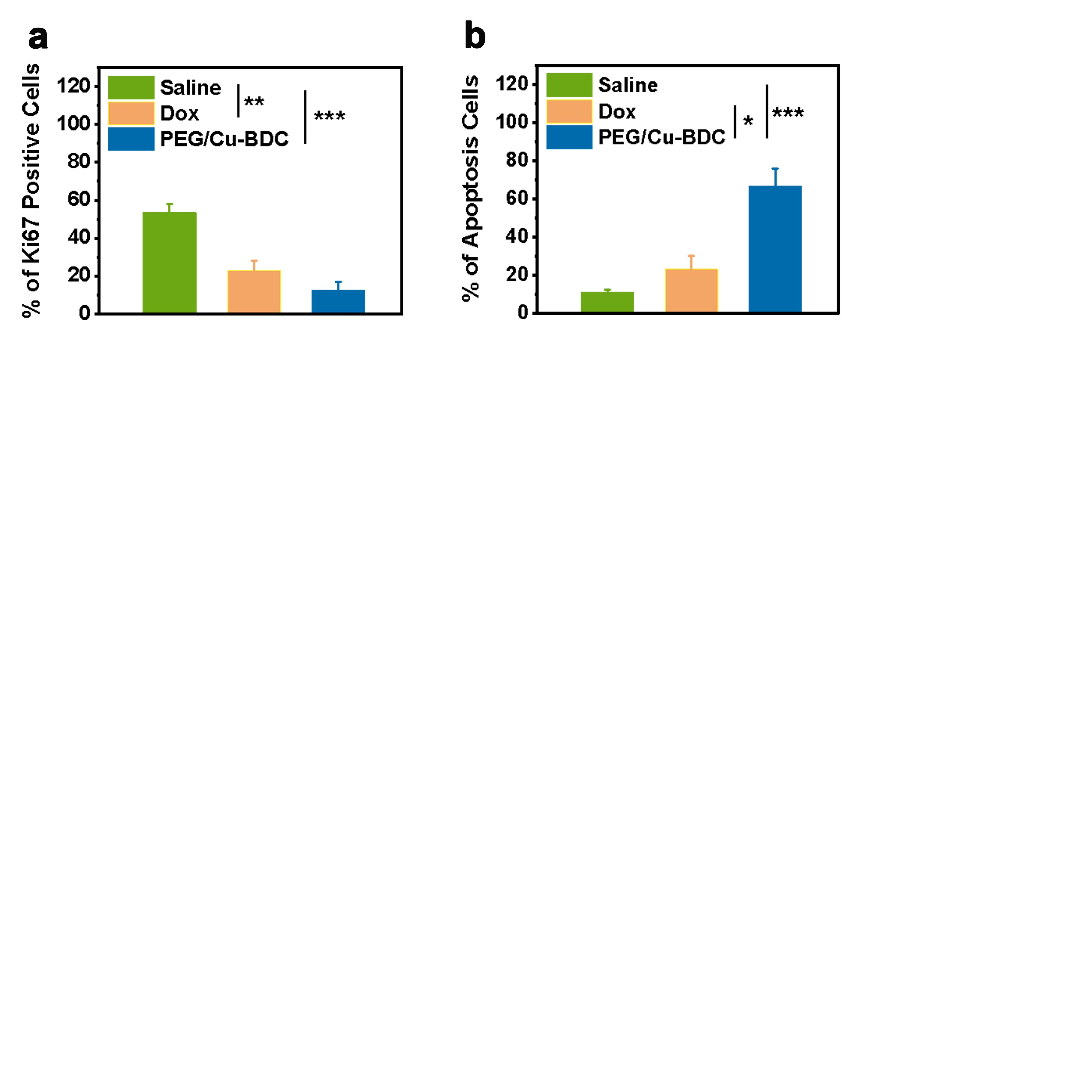


**Figure S9.** Quantitative analysis of Ki-67 and apoptosis-positive tumor cells of MDA-MB-231 tumor-bearing mice in different treatment groups.
